# Incorporating biological and clinical insights into variant choice for Mendelian randomisation: examples and principles

**DOI:** 10.1136/egastro-2023-100042

**Published:** 2024-01-22

**Authors:** Stephen Burgess, Héléne Toinét Cronjé

**Affiliations:** 1 MRC Biostatistics Unit, University of Cambridge, Cambridge, UK; 2 Cardiovascular Epidemiology Unit, Department of Public Health and Primary Care, University of Cambridge, Cambridge, UK; 3 Health Analytics, Lane Clark & Peacock LLP, London, UK; 4 Department of Public Health, Section of Epidemiology, University of Copenhagen, København, Denmark

## Abstract

Mendelian randomisation is an accessible and valuable epidemiological approach to provide insight into the causal nature of relationships between risk factor exposures and disease outcomes. However, if performed without critical thought, we may simply have replaced one set of implausible assumptions (no unmeasured confounding or reverse causation) with another set of implausible assumptions (no pleiotropy or other instrument invalidity). The most critical decision to avoid pleiotropy is which genetic variants to use as instrumental variables. Two broad strategies for instrument selection are a biologically motivated strategy and a genome-wide strategy; in general, a biologically motivated strategy is preferred. In this review, we discuss various ways of implementing a biologically motivated selection strategy: using variants in a coding gene region for the exposure or a gene region that encodes a regulator of exposure levels, using a positive control variable and using a biomarker as the exposure rather than its behavioural proxy. In some cases, a genome-wide analysis can provide important complementary evidence, even when its reliability is questionable. In other cases, a biologically-motivated analysis may not be possible. The choice of genetic variants must be informed by biological and functional considerations where possible, requiring collaboration to combine biological and clinical insights with appropriate statistical methodology.

## Introduction

Mendelian randomisation is an epidemiological approach for making causal inferences from observational data based on genetic variants.^1 2^ The choice of which genetic variants to include in a Mendelian randomisation analysis is fundamental to the validity of the analysis.^3^ Genetic variants are assumed to act analogously to randomisation in a clinical trial, affecting the exposure of interest, but not affecting traits on alternative causal pathways to the outcome, and not being associated with the outcome via confounding pathways.^4 5^ These assumptions form the definition of an instrumental variable.^6^ The instrumental variable assumptions imply that the genetic variants will only be associated with the exposure and with any traits on causal pathways from the genetic variants to the exposure and beyond. They would be violated if the genetic variant influenced the outcome by a causal pathway not acting via the exposure, or if the genetic variant was associated with another variable that affects the outcome.

**Figure 1:**
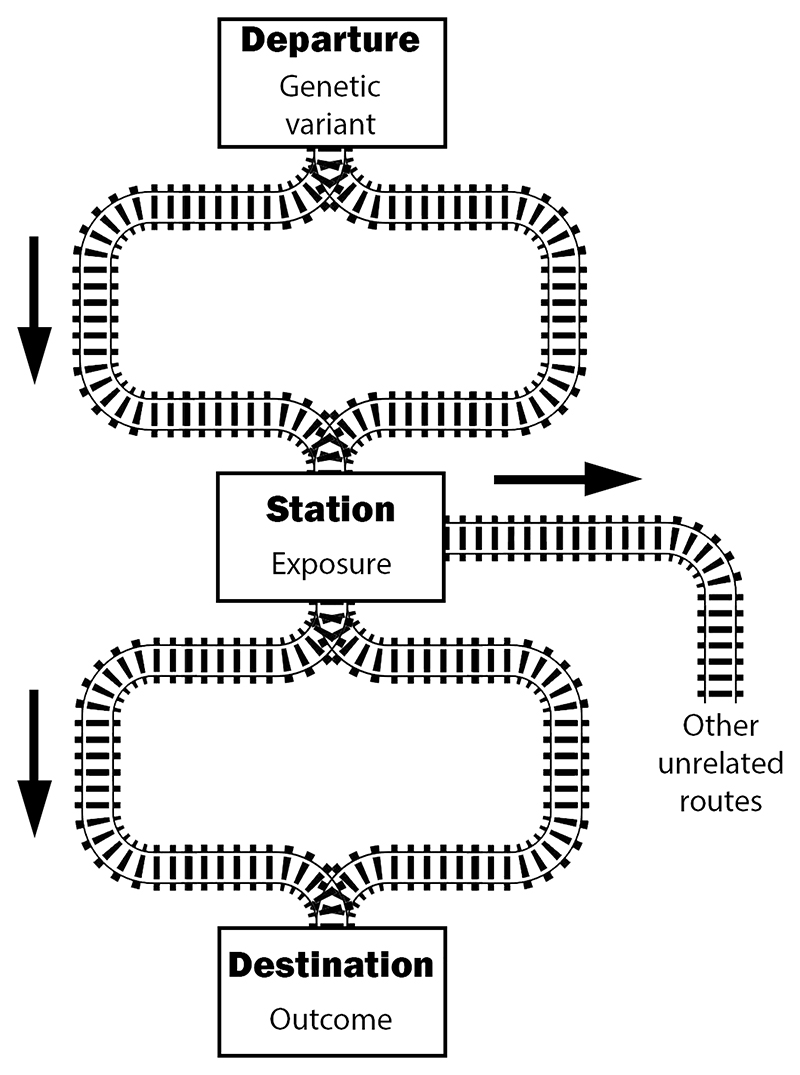
Causal assumptions of Mendelian randomisation illustrated as a railway network. The instrument variable assumptions imply that all trains leaving the departure station (genetic variant) and arriving at the destination (outcome) must pass through the exposure station. They can take different routes, reflecting different causal pathways, and there can be some pathways from the exposure to unrelated destinations. But the exposure station must be a station stop on all routes from the variant to the outcome.

If we imagine causal pathways as a railway network from the genetic variant to the outcome, all trains must pass through the exposure (figure 1). There can be branching pathways down the track from the exposure, or even before reaching the exposure, but the exposure must be a station stop on all routes to the outcome.

In general, the plausibility of satisfying the instrumental variable assumptions will be greater when the distance between the genetic variants and the exposure is short, and when the functional relevance of the genetic variants to the exposure is clearly understood.^7^ Hence, Mendelian randomisation analyses will be most reliable when the exposure is the direct product of a coding gene region (such as a protein), and the analysis is restricted to using variants from that gene region.^8^ Such analyses, particularly for proteins that are the target for pharmacological intervention, have been labelled as ‘*cis*-Mendelian randomisation analyses’, as they use *cis*-genetic variants.^9^ Alternatively, genetic variants in a Mendelian randomisation analysis may not be in the gene region that encodes the exposure, but they may encode key regulators of the exposure levels; hence, the variant choice can be biologically motivated even if it is not a *cis*-Mendelian randomisation analysis.

### Biologically motivated versus genome-wide Mendelian randomisation analyses

There are several advantages of biologically motivated Mendelian randomisation analyses (and *cis*-Mendelian randomisation analyses, in particular) over genome-wide Mendelian randomisation analyses (eg, analyses using variants associated with the exposure from any gene region across the genome). Aside from greater plausibility of the instrumental variable assumptions, a *cis*-Mendelian randomisation analysis can be specifically relevant to a particular intervention pathway.^10^ For example, a Mendelian randomisation analysis to consider the effect of blood pressure lowering using variants in the *ADRB1* gene region is particularly relevant for assessing the effect of beta-blockers, as *ADRB1* encodes the beta-1 adrenergic receptor, which is inhibited by beta-blockers. Similarly, a Mendelian randomisation analysis using variants in the *ACE* gene region is particularly relevant for assessing the effect of ACE inhibitors, as *ACE* encodes the ACE protein.^11^ It is plausible that different blood pressure-lowering mechanisms have different effects on various outcomes.^12^ Insights into mechanistic nuances within broader drug indication categories (eg, antihypertensive drugs) allow clinicians to appropriately assign treatments to specific population subgroups based on individualised treatment goals and risk profiles. One example is the differential risk relating to offspring birth weight reported by a Mendelian randomisation analysis comparing the predicted effects of beta-blockers versus calcium-channel inhibitors to manage blood pressure during pregnancy.^13^

However, there are also potential advantages of genome-wide Mendelian randomisation analyses. By considering variants from multiple gene regions, the consistency of results can be assessed.^14^ If multiple genetic variants influencing the same exposure are associated with the outcome in the same direction (ie, the exposure-increasing allele is consistently associated with higher or lower risk of the outcome), this provides supporting evidence for a causal effect of the exposure on the outcome. For example, genetic variants associated with higher low-density lipoprotein (LDL) cholesterol in multiple gene regions are consistently associated with increased risk of coronary heart disease (CHD).^15^

On the contrary, if genetic variants associated with the same exposure are associated with the outcome in different directions, this can be an indication of mechanism-specific effects. For example, genetic variants associated with LDL-cholesterol have differing associations with risk of gallstones.^16^ In particular, for the *HMGCR* gene region (which encodes the target of statin drugs), variants associated with lower plasma LDL-cholesterol are associated with lower risk of gallstones, whereas for the *ABCG5/ABCG8* gene region, variants associated with lower plasma LDL-cholesterol are associated with higher risk of gallstones. This potentially reflects differences between mechanisms that reduce cholesterol systemically, and those that affect cholesterol transportation, hence reducing circulating LDL-cholesterol but increasing biliary cholesterol (which is the key risk factor for developing gallstones).

In this review, we will consider examples where Mendelian randomisation analyses have been performed using different instrument selection strategies and compare results from these analyses. We compare biologically motivated versus genome-wide strategies for variant selection, we consider positive controls for the selection of variants, and we compare variant selection based on associations with a circulating biomarker versus a behavioural phenotype.

### C reactive protein: variants in the protein coding gene region

**Figure 2:**
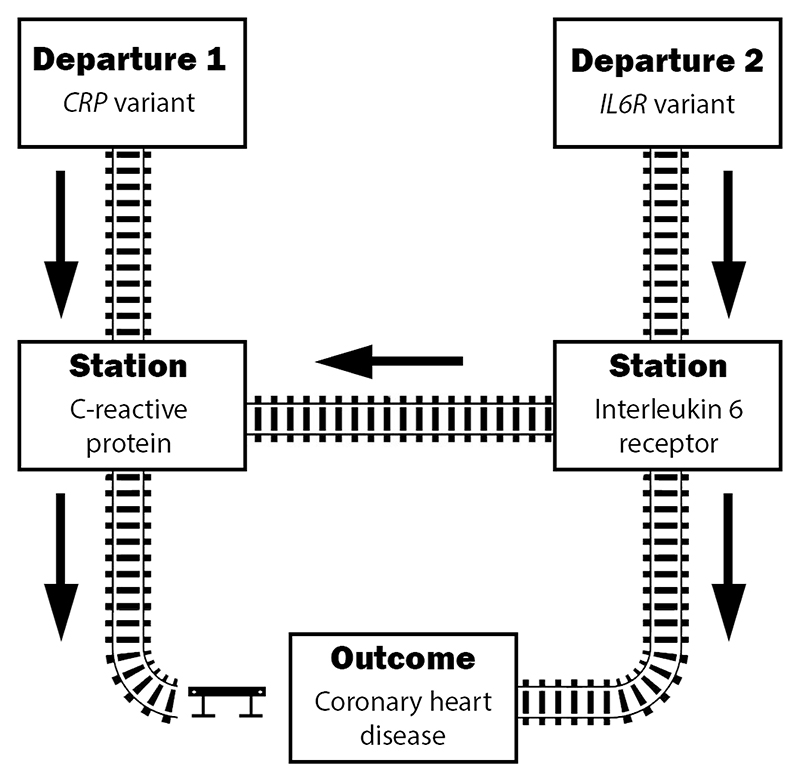
Railway network illustrating pathways linking inflammatory traits to coronary heart disease. Trains leaving from departure station 1 (variant in *CRP* gene region) pass first through C reactive protein, whereas trains leaving from departure station 2 (variant in *IL6R* gene region) pass first through interleukin-6 receptor and then branch out either to C reactive protein or directly to the outcome. As variants in the *CRP* gene region do not associate with the outcome that railway line is blocked by the buffer stop/stopblock. Variants in the *IL6R* gene region do associate with the outcome, implying that there is a functioning route from departure station 2 to the outcome. However, due to the buffer stop on the route from C reactive protein to the outcome, the effect of interleukin-6 receptor on the outcome must be direct and not via C reactive protein, and hence interleukin-6 receptor is a causal risk factor for coronary heart disease, but C reactive protein is not.

C reactive protein (CRP) is an acute phase reactant that forms part of the body’s inflammatory response. CRP is encoded by the *CRP* gene and is expressed in the liver in response to upstream cytokines, most notably interleukin-6. Mendelian randomisation analyses using variants in the *CRP* gene region to instrument variation in circulating CRP levels have shown null associations with CHD risk, suggesting that CRP is not a causal risk factor for CHD.^17 18^ In contrast, Mendelian randomisation analyses using variants associated with circulating CRP in the interleukin-6 receptor gene region *(IL6R)* have shown inverse associations with CHD risk, suggesting that CRP might be protective against CHD.^19 20^ However, genetic variants in the *IL6R* gene region primarily affect interleukin-6 receptor, which is upstream of CRP in the inflammatory response pathway.^21^ Therefore, it is likely that interleukin-6 receptor rather than CRP is the causal risk factor for CHD (figure 2).

Indeed, a Mendelian randomisation analysis of CHD that instruments CRP using a genome-wide approach gives contradictory answers, with some genetic predictors of increased CRP levels being associated with lower risk of CHD, and others being associated with higher risk of CHD.^22^ Hence, not all genome-wide significant predictors of circulating CRP can be valid instrumental variables. We would opine that the Mendelian randomisation analysis restricted to variants in the *CRP* gene region is the most relevant assessment of the causal effect of CRP, and hence there is no compelling evidence of a causal effect of CRP on CHD risk. In this case, the genome-wide analysis has limited utility. It only demonstrates the lack of consistency in the effect of inflammation on CHD risk, implying that there are mechanism-specific or pleiotropic effects.

### Alcohol consumption: variants affecting exposure metabolism

Mendelian randomisation analyses in East Asian populations have generally used variants in the *ADH1B* and *ALDH2* gene regions, as these gene regions contain variants strongly associated with alcohol consumption, and their functional relevance to alcohol biology is clear. Alcohol dehydrogenase (encoded by *ADH1B*) is the enzyme responsible for oxidising alcohol to acetaldehyde—the toxic compound associated with discomfort brought on by consuming alcohol—which is further metabolised to acetate by the enzyme aldehyde dehydrogenase (encoded by *ALDH2*). Because of the strong relationship between genetic variation in these regions and the ability to metabolise and tolerate alcohol, these variants strongly relate to (and are plausible instrumental variables for) alcohol consumption behaviour.^23^ Notably, in European-ancestry populations, commonly occurring variants within these same gene regions do not affect alcohol metabolism to the same extent as in East Asian populations and are, therefore, less suitable instrumental variables for alcohol consumption. Some analyses in European populations have used a limited set of biologically selected variants^24^; others have used all variants associated with alcohol consumption^25^ or alcohol use disorder^26^ at a genome-wide level of statistical significance. The former approach has more specificity, whereas the latter approach potentially has greater statistical power.

This point was illustrated in a Mendelian randomisa-tion study reporting on the effect of alcohol consumption on the risk of stroke in a European-ancestry population using three strategies for genetic instrument selection.^25^ The effect estimate when alcohol consumption was instrumented using only a single *ADH1B* variant (rs1229984), which was 1.33 (95% CI 1.06 to 1.62), compared with 1.26 (1.08 to 1.47) when using 93 genome-wide significant variants (excluding rs1229984); and 1.27 (1.12 to 1.45) when using 94 genome-wide significant variants (including rs1229984). These estimates are ORs per 1 SD increase in log transformed genetically predicted number of alcohol units per week. In this case, estimates were consistent across approaches, with use of more variants corresponding to narrower CIs. When more than one plausible instrument selection strategy is available, we advise presenting estimates from all approaches, as it allows the reader to judge the consistency and strength of evidence.

### Alcohol consumption: sensitivity analyses to assess instrument validity

When performing Mendelian randomisation analyses for the effect of alcohol consumption, several sensitivity analyses can be performed to assess the validity of findings. In parts of East Asia, alcohol consumption levels vary strongly between men and women.^27^ If women do not drink alcohol in a specific population, then genetic variants that influence alcohol consumption should be associated with downstream consequences of alcohol consumption for men but not for women in that population. For example, when considering alcohol-related variants to investigate potential effects on blood pressure in a South Korean population, genetic associations were evident in men but not in women, consistent with a causal effect of alcohol consumption on blood pressure.^28^ In this example, women act as a negative control population. If genetic associations with outcomes were similar in men and women, then it would not be reasonable to claim that the associations are due to the causal effect of alcohol consumption.

Similarly, genetic associations with oesophageal cancer risk have been compared in alcohol abstainers, light drinkers and heavy drinkers. Genetic associations for alcohol variants were around three times stronger in heavy drinkers compared with light drinkers.^29^ Again, this is consistent with alcohol being the causal risk factor driving these genetic associations. Some caution is required, however, as the exposure is a collider in the standard instrumental variable graph, as it is a common effect of the genetic variants and exposure—outcome confounders. Hence, stratification on alcohol status will lead to the genetic associations being distorted by collider bias.^30^ In this case, it is implausible that collider bias alone would account for the large differences in genetic associations in the alcohol consumption groups.^31^ Caution should be expressed when stratifying or otherwise adjusting for a variable, that is a potential collider, as small differences in associations may be attributable to collider bias. We note that sex is not a downstream consequence of autosomal genetic variants, hence stratifying associations on sex (as in the example above) will not lead to collider bias.

In the context of Mendelian randomisation, pleiotropy (sometimes called horizontal pleiotropy) refers to the association of genetic variants with multiple traits on separate causal pathways. Pleiotropy is a threat to the validity of Mendelian randomisation analyses. A valuable technique to assess the impact of pleiotropy is multivariable Mendelian randomisation.^32 33^ While standard Mendelian randomisation assesses whether genetically predicted levels of an exposure are associated with the outcome in a univariable model, multivariable Mendelian randomisation assesses whether genetically predicted levels of an exposure are associated with the outcome in a multi-variable model with adjustment for genetically predicted values of related traits. An association in standard Mendelian randomisation is indicative (under the instrumental variable assumptions) of a causal effect of the exposure on the outcome. Similarly, an association in multivariable Mendelian randomisation is indicative of a direct causal effect of the exposure on the outcome, independent of other traits included in the multivariable model.^34^

For alcohol consumption, it is plausible that genetic variants which influence alcohol consumption also influence smoking behaviour, either through biological pleiotropy (variants affecting propensity to take addictive substances) or downstream effects of alcohol consumption (including the social effect of visiting licenced establishments). A multivariable Mendelian randomisation analysis adjusting for genetically predicted smoking behaviour is able to verify whether findings are due to direct effects of alcohol consumption, and not indirect or pleiotropic effects acting via smoking behaviour. A Mendelian randomisation analysis examining the effect of alcohol consumption on various gastrointestinal diseases revealed an association between genetically predicted alcohol consumption and risk of duodenal ulcer.^35^ However, attenuation on adjustment for genetically predicted smoking initiation suggests that this effect, at least in part, could be mediated by smoking behaviour.

### Vitamin D supplementation: variants affecting synthesis and metabolism

In humans, vitamin D is mostly obtained from diet and supplementation and activated by various biological processes that are catalysed by different enzymes. Several Mendelian randomisation analyses investigating the impact of vitamin D insufficiency on health and disease risk have used variants from a small number of gene regions with direct relevance to vitamin D synthesis or metabolism.^36^ For example, a previous investigation reported concordant associations with the risk of multiple sclerosis across four genetic variants in different gene regions, each related either to the synthesis, transport or metabolism of vitamin D.^37^ The consistency between these associations provides additional strength of evidence compared with an analysis only using a single variant.

As genome-wide association studies have expanded in size, more variants associated with vitamin D metabolites (in particular, 25-hydroxyvitamin D) have been discovered.^38 39^ However, several of these variants additionally associate with other risk factors, such as LDL-cholesterol.^40^ While the biologically informed choice of variants affords more specificity, the genome-wide choice of variants could provide greater statistical power. A potential resolution is to restrict the analysis to variants in biologically relevant gene regions, but to select multiple variants from each gene region to increase the proportion of variance in the exposure explained by the instrument, and hence increase statistical power.^41^

For vitamin D, there is less motivation to consider a genome-wide instrument compared with alcohol consumption in European populations, as: (1) variants in biologically relevant gene regions explain around 4% of the variance in circulating 25-hydroxyvitamin D levels, meaning that such analyses already have adequate statistical power,^41^ and (2) consistency between results based on different gene regions can be assessed even for the biologically informed selection strategy. This differs from the example of alcohol consumption above, as the *ADH1B* alcohol variant explains around 0.2% of the variability in alcohol intake in European-ancestry populations.^42^

We note in passing that, while 4% does not sound like a large percentage, an intervention in a randomised trial does not explain 100% of the variance in an exposure. For example, vitamin D supplementation in the Vitamin D and Omega-3 Trial (VITAL) trial only accounted for 26% of the variance in 25-hydroxyvitamin D levels.^43^ However, while the limited proportion of variance explained restricts statistical power to detect a causal effect, it does not invalidate causal inferences either in a clinical trial or a Mendelian randomisation investigation.

### Estradiol: validating instruments using a positive control

The protective effect of oestrogen on breast and endometrial cancer is well attested from studies into hormone replacement therapy^44^ and use of oestrogen-containing contraceptive pills.^45 46^ Several genetic variants are associated with circulating levels of estradiol, a specific oestrogen steroid hormone. However, some of these variants are not associated with breast and endometrial cancer, and so the extent to which these variants mimic interventions that alter oestrogen levels is unclear. To investigate the potential impact of therapies aimed at increasing oestrogen levels on different site-specific cancers, breast cancer (and/or endometrial cancer) can be used as positive controls for the selection of genetic variants into the instrumental variable.

A Mendelian randomisation study implementing this approach ultimately used only a single genetic variant to instrument oestrogen raising, as all other estradiol-associated variants were not associated with the positive control outcomes.^47^ No associations with other site-specific cancers were observed, providing some evidence that the impact of estrogen-raising interventions on cancer outcomes is restricted to breast and endometrial cancers. A limitation of this analysis is statistical power; as only one variant was included in the analysis, power to detect a causal effect was limited, and the consistency of results across variants could not be assessed.

In addition to positive controls, analysts may make use of negative controls in the selection of genetic variants. For example, prepubertal asthma was used as a negative control outcome in a Mendelian randomisation study to investigate the effects of age at puberty on asthma.^48^ Similarly, when investigating the effects of adult-onset asthma, childhood asthma could be used as a negative control for the selection of variants to ensure that the genetic variants only affect the outcome via adult-onset asthma. We note that both positive and negative controls (including control outcomes, control exposures, control populations and control time periods) can be used in many ways,^49^ including in the selection of genetic variants and the validation of results based on the selected variants.

### Plasma caffeine levels versus coffee consumption: different variants, different causal questions

To investigate the impact of coffee as an exposure, complementary instrumental variable selection strategies could be considered. Genetic variants could be selected based on their associations with circulating plasma caffeine levels, or behavioural estimates of coffee consumption (eg, number of cups consumed per day).^50^ Alternatively, variants could be selected based on biological considerations; only variants in gene regions relating to caffeine metabolism could be considered.^51^ While it would be natural to think that genetic variants associated with greater coffee consumption would also be associated with higher circulating caffeine levels, this is not true.^52^ Some genetic variants that cause individuals to metabolise caffeine less rapidly are associated with increased caffeine levels, but decreased coffee consumption, as these individuals need to drink less coffee to get the same physiological effect. A similar relationship has been observed for smoking, as individuals who metabolise nicotine more quickly tend to smoke more cigarettes per day.^53^

If the causal question of interest relates to the consumption of hot liquids (say we are investigating the impact of coffee on oesophageal cancer),^54^ then coffee consumption may be the true risk factor of interest and, therefore, the one we would instrument. However, if the causal question of interest relates to the effect of caffeine, then using variants that are associated with coffee consumption could be misleading. Differing Mendelian randomisation estimates for the effects of coffee on body mass index depending on the choice of variants (genome-wide or biologically-motivated) and exposure (caffeine levels or coffee consumption)^52^ illustrate the importance of carefully defining the causal effect of interest,^55^ and ensuring an appropriate choice of genetic instrumental variables to match the causal question.

### Should only biologically motivated Mendelian randomisation analyses be performed?

While all recommendations should be weighed carefully for any specific application, in general, we would encourage researchers to focus on biologically motivated strategies for variant selection in Mendelian randomisation analyses where possible. Genome-wide analyses can provide supporting evidence, but often add more noise to the analysis than reliable signal. Examples include the inconsistent Mendelian randomisation evidence in support or disagreement of adiponectin being a causal contributor to cardiometabolic disease depending on whether it is instrumented using variants in its coding gene region (*ADIPOQ*) only^56^ or all predictors across the genome.^57^ While genome-wide analyses can have increased power, there is greater potential for instrument invalidity due to the increased number of genetic variants.

If there is strong consistency in results across variants in a genome-wide analysis, then strength of evidence for a causal conclusion is increased. However, a significant inverse-variance weighted estimate may be obtained if (say) 55% of the included variants are associated with an increased risk of the outcome compared with the remaining 45% that are associated with a decreased risk. Such a result would not reflect the consensus needed to provide reliable evidence of a causal effect. Although the use of robust statistical methods can help to distinguish cases where a causal effect is evidenced by one or two variants versus by the majority of the variants, most robust methods for Mendelian randomisation make similar assumptions for consistent estimation,^58^ and so should not be regarded as foolproof.

As genome-wide association studies increase in size, they will discover more variants associated with potential exposures. There is a temptation to incorporate all these variants into Mendelian randomisation analyses. A better approach is to focus on biologically relevant gene regions and select (if available) multiple variants from these gene regions that predict independent variability in the exposure (with summarised data, accounting for correlations between variants if necessary),^59^ to maximise the proportion of variance in the exposure explained, and hence maximise power of the Mendelian randomisation analysis while maintaining specificity.

One potential conclusion from this recommendation is that only Mendelian randomisation analyses with biologically relevant genetic variants should be attempted. We would not agree with this conclusion. For a given exposure, if there is a choice between a biologically relevant set of variants and a genome-wide set of variants, then we would generally prefer the analysis based on the biologically relevant set of variants. However, there are many exposures worth investigating for which a biologically relevant set of variants is not available. Although inferences from Mendelian randomisation analyses in such cases will be questionable, other sources of evidence are also likely to be imperfect, and so a Mendelian randomisation analysis can provide important evidence supporting or refuting a causal claim, even if that evidence by itself is not conclusive.

For example, sleep duration is known risk factor for a wide range of diseases.^60 61^ However, biological mechanisms affecting sleep duration, and hence genetic variants that are candidate instruments for biologically-motivated Mendelian randomisation analyses, are poorly understood.^62^ Randomised trials investigating the long-term impact of sleep duration on health outcomes are infeasible, and observational analyses are likely subjected to unmeasured confounding and reverse causation. Hence, even though findings from Mendelian randomisation analyses with sleep duration as the exposure will not be ‘beyond reasonable doubt’ as to their validity, they are an important source of evidence on the long-term effect of sleep duration on health outcomes.

## Conclusion

**Figure 3:**
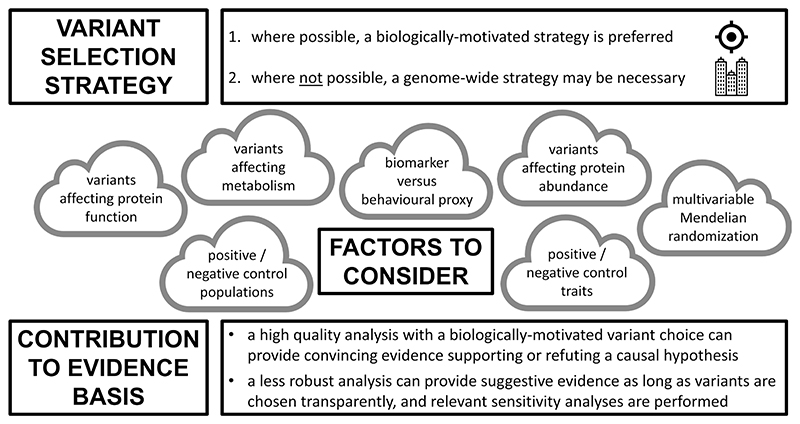
Central illustration of factors influencing variant choice in Mendelian randomisation.

A Mendelian randomisation investigation should never be approached from a purely statistical perspective (figure 3). The choice of genetic variants must be informed by biological and functional considerations where possible, and statistical methods for assessing instrument validity should be accompanied by other assessments of the robustness of findings, such as the use of positive and negative controls.^63^ Not all Mendelian randomisation analyses will be equivalent in terms of the strength of evidence provided, but validity can be enhanced by meaningful collaboration across scientific disciplines, incorporating biological and clinical insights together with appropriate statistical methodology.
