# Supplementary material for: Incorporating biological and clinical insights into variant choice for Mendelian randomisation: examples and principles
Source: eGastroenterology. 2024 Jan 19;2(1):e100042. doi: 10.1136/egastro-2023-100042 (PMC7615644; doi:10.1136/egastro-2023-100042)
Supplement: Supplementary file 1 [file egastro-2023-100042.reviewer_comments.pdf]

PEER REVIEW HISTORY

eGastroenterology publishes all reviews undertaken for accepted manuscripts. Reviewers are asked to complete a checklist review form and are provided with free text boxes to elaborate on their assessment. These free text comments are reproduced below.

ARTICLE DETAILS

**TITLE (PROVISIONAL)**	Incorporating biological and clinical insights into variant choice for Mendelian randomization: examples and principles
**AUTHORS**	Stephen Burgess, Héléne Toinét Cronjé

VERSION 1 – REVIEW

**REVIEWER**	Alisa Kjaergaard
**REVIEW RETURNED**	19-Oct-2023

**GENERAL COMMENTS**	This manuscript is a pleasure to read. It summarizes concisely and clearly important issues in MR studies.<br>I suggest adding a paragraph to the "Alcohol consumption: variants affecting exposure metabolism" section regarding the sex differences observed in Asian populations due to a low level of alcohol consumption in women and how this this is analogous to the RCT framework and underlines the assumption that the variant influences outcomes only through the exposure i.e. alcohol intake. This is similar to variants associated with smoking in smokers and non-smokers, which should also be mentioned.<br>In addition to positive controls, the authors should add a section on the importance of using negative controls as well. This is particularly important since the "negative controls", and reference 53 are only mentioned in the Conclusion.

**REVIEWER**	Chen, Lanlan
**REVIEW RETURNED**	23-Oct-2023

**GENERAL COMMENTS**	This is a well-written paper which aims to address the most important issue in Mendelian randomization --- choosing suitable genetic variants as instrumental variables. This paper is clear and informative and will be a good guidance for performing MR study. I only have several minor comments:<br><br>1. The authors mentioned "negative control" in the Discussion and it is a important part in MR analysis. I recommend to add one paragraph to illustrate the "negative control", just after the "positive control".<br>2. This paper is clear, but the readers would benefit from a checklist displaying the items relevant to selecting instruments.<br>3. Since eGastroenterology is a journal focusing on digestive diseases, it is recommended to add 1~2 examples related to digestive diseases.

VERSION 1 – AUTHOR RESPONSE

Response to reviewers for "Incorporating biological and clinical insights into variant choice for Mendelian randomization: examples and principles": EGASTRO-2023-100042

We would like to express thanks to the reviewers and editor for their time and comments. Replies to points are indicated by angle brackets, and changes to the paper as a result of these comments are clearly indicated. We have numbered the comments for reference as A1, A2 for the first reviewer; and B1, B2, B3 for the second reviewer.

Reviewer 1: Alisa Kjaergaard, Aarhus Universitet

A0. This manuscript is a pleasure to read. It summarizes concisely and clearly important issues in MR studies.

> We thank the reviewer for her positive view of this manuscript, and for her constructive suggestions. <

A1. I suggest adding a paragraph to the “Alcohol consumption: variants affecting exposure metabolism” section regarding the sex differences observed in Asian populations due to a low level of alcohol consumption in women and how this this is analogous to the RCT framework and underlines the assumption that the variant influences outcomes only through the exposure i.e. alcohol intake. This is similar to variants associated with smoking in smokers and non-smokers, which should also be mentioned.

> This is a helpful suggestion. We have added a section to the manuscript entitled “Alcohol consumption: sensitivity analyses to assess instrument validity” (pages 4-5) in which we discuss the use of sex-stratified genetic estimates, estimates stratified on the exposure level, and multivariable Mendelian randomization.

> While we appreciate the suggestion to consider genetic associations in smokers and nonsmokers, we have instead discussed genetic associations in non-drinkers, light drinkers, and heavy drinkers, so as to keep consistent the example of alcohol consumption. We have clarified the difference between stratification on sex (which cannot be a collider) and alcohol consumption level (which is likely to be a collider). <

A2. In addition to positive controls, the authors should add a section on the importance of using negative controls as well. This is particularly important since the “negative controls”, and reference 53 are only mentioned in the Conclusion.

> We appreciate this suggestion, and have added an example where a negative control can be used for variant selection. We note that the use of positive and negative controls discussed in the conclusion is slightly different – such variables can be used to select amongst variants, but they can also be used to validate the Mendelian randomization analysis.

> We have added to the manuscript: “In addition to positive controls, analysts may make use of negative controls in the selection of genetic variants. For example, pre-pubertal asthma was used as a negative control outcome in a Mendelian randomization study to investigate the effects of age at puberty on asthma. Similarly, when investigating the effects of adultonset asthma, childhood asthma could be used as a negative control for the selection of variants to ensure that the genetic variants only affect the outcome via adult-onset asthma. We note that both positive and negative controls (including control outcomes, control exposures, control populations, and control time-periods) can be used in many ways, including in the selection of genetic variants, and the validation of results based on the selected variants.” (page 6).

Reviewer 2: Dr. Lanlan Chen, The First Hospital of Jilin University

B0. This is a well-written paper which aims to address the most important issue in Mendelian randomization --- choosing suitable genetic variants as instrumental variables. This paper is clear and informative and will be a good guidance for performing MR study.

> We thank the reviewer for his positive view of this manuscript, and for his constructive suggestions. <

I only have several minor comments:

B1. The authors mentioned “negative control” in the Discussion and it is a important part in MR analysis. I recommend to add one paragraph to illustrate the “negative control”, just after the “positive control”.

> We appreciate this suggestion. Please see the response to reviewer point A2, which also made a similar point. <
B2. This paper is clear, but the readers would benefit from a checklist displaying the items relevant to selecting instruments.
> We appreciate this suggestion. We have added a visual summary (central illustration) that summarizes the content of the manuscript as Figure 3. <
B3. Since eGastroenterology is a journal focusing on digestive diseases, it is recommended to add 1~2 examples related to digestive diseases.
> This is a good suggestion. We have already included an example about the effect of cholesterol on gallstone disease, which begins: "For example, genetic variants associated with LDL-cholesterol have differing associations with risk of gallstones" (page 3), and an example on the effect of coffee on oesophageal cancer (page 7). We have now added examples and references discussing the effect of alcohol consumption on oesophageal cancer (page 4) and on various gastrointestinal diseases (page 5). <

VERSION 2 – REVIEW

**REVIEWER**	Kjaergaard , Alisa
**REVIEW RETURNED**	09-Nov-2023

**GENERAL COMMENTS**	Thank you for addressing the comments so nicely.

**REVIEWER**	Chen, Lanlan
**REVIEW RETURNED**	13-Nov-2023

**GENERAL COMMENTS**	This revision is well-written and I have no other comments.
